# Continuous spinal anaesthesia versus ultrasound-guided combined psoas compartment-sciatic nerve block for hip replacement surgery in elderly high-risk patients: a prospective randomised study

**DOI:** 10.1186/1471-2253-14-99

**Published:** 2014-11-05

**Authors:** Mehmet Aksoy, Aysenur Dostbil, Ilker Ince, Ali Ahiskalioglu, Hacı Ahmet Alici, Ali Aydin, Osman Ozgur Kilinc

**Affiliations:** Department of Anaesthesiology and Reanimation, Faculty of Medicine, Ataturk University, Erzurum, Turkey; Department of Orthopedics and Traumatology, Faculty of Medicine, Ataturk University, Erzurum, Turkey

**Keywords:** Anaesthesia technique, Hip surgery, High-risk patient, Continuous spinal anaesthesia, Combined psoas compartment-sciatic nerve block

## Abstract

**Background:**

Our aim is to compare the hemodynamic effects of combined psoas compartment-sciatic nerve block (PCSNB) with continuous spinal anaesthesia (CSA) in elderly high-risk patients undergoing hip replacement surgery.

**Methods:**

Seventy patients over the age of 60 with ASA III or IV physical status were randomly allocated to two groups: In the PCSNB group, ultrasound-guided psoas compartment block was performed with modified Winnie technique using 30 mL of 0.25% bupivacaine with 1:200.000 epinephrine (5 μgr/mL) and iliac crest block was performed using the same local anaesthetic solution (5 mL). All patients in the PCSNB group needed continuing infusion of propofol (2 mg/kg/h) during operation. In the CSA group, CSA was performed in the L_3_-L_4_ interspaced with the patient in lateral decubitus position using 2.5 mg of isobaric bupivacaine 0.5%. When sensory block was not reached to the level of T_12_ within 10 minutes in the CSA group, additional 2.5 mg of isobaric bupivacaine 0.5% was administered through the catheter at 5-min intervals by limiting the total dose of 15 mg until a T_12_ level of the sensory block was achieved.

**Results:**

The PCSNB group had significantly higher mean arterial blood pressure values at the beginning of surgery and at 5^th^, 10^th^ and 20^th^ minutes of surgery compared to the CSA group (P =0.038, P =0.029, P =0.012, P =0.009 respectively). There were no significant differences between groups in terms of heart rate and peripheral oxygen saturation values during surgery and the postoperative period (P >0.05). Arterial hypotension required ephedrine was observed in 13 patients in the CSA and 4 patients in the PCSNB group (P =0.012).

**Conclusions:**

CSA and PCSNB produce satisfactory quality of anaesthesia in elderly high-risk patients with fewer hemodynamic changes in PCSNB cases compared with CSA cases.

**Trial registration:**

Australian New Zealand Clinical Trials Registry: ACTRN12614000658617, Registered 24 June 2014.

## Background

Hip replacement surgery is common among elderly patients. These patients have increased risk of perioperative mortality and morbidity due to additional co-morbidities such as cardiac, endocrine, renal, cerebral and respiratory diseases [1]. It has been shown that the use of regional anaesthetic methods during hip replacement surgery reduces intraoperative blood loss and the risk of postoperative deep venous thrombosis [2, 3]. Also, lower odds for the need of postoperative critical care services were reported in the use of neuraxial anaesthesia compared with general anaesthesia in total hip arthroplasty patients [4]. Despite these advantages, regional anaesthetic methods are difficult technically and there is a high-risk of failure in the implementation of these methods [3].

Continuous spinal anaesthesia (CSA) provides extension of blockage during surgery and versatile pain management during the postoperative period via an indwelling catheter allowed intermittent injection of local anaesthetic into the subarachnoid space. Better cardiovascular stability with a smaller dose of local anaesthetic and shorter surgery onset time were reported in CSA compared with combined spinal epidural block, among patients undergoing major hip, femoral or knee surgery [5]. Conversely, CSA may lead to adverse hemodynamic changes due to the extent of sympathetic blockade which is affected by existing cardiac disease and intravascular volume status. Also, CSA was found to be associated with a high incidence of post-dural puncture headache (PDPH) [6]. This undesirable side effect was reduced by using smaller needles and microcatheters for the block procedure [7].

Combined psoas compartment-sciatic nerve block (PCSNB) provides adequate anaesthesia for repair of hip fractures and causes more limited sympathectomy without bladder paralysis [8]. However, this technique has some undesirable complications such as epidural spread, total spinal anaesthesia, retroperitoneal hematoma and renal puncture [3]. It was reported that the use of ultrasound imaging when performing a PCSNB facilitates correct positioning of the needle and may reduce the incidence of undesirable side effects [9]. Also, ultrasound technology provides the actual identification of target organs, the visualization of spread of the injected local anaesthetic in real-time, a decrease in the number of attempts and improved block quality [10].

We hypothesised that PCSNB technique could be more favourable for elderly high-risk patients undergoing hip replacement surgery due to limited sympathectomy. Also, we thought that the safety of PCSNB would be enhanced by ultrasound guidance. Therefore, this prospective randomised study was designed to compare the hemodynamic effects and anaesthesia quality of PCSNB with CSA in elderly high-risk patients undergoing hip replacement surgery.

## Methods

This prospective randomised study was performed at Department of Anaesthesiology and Reanimation, Ataturk University, Medical Faculty, Erzurum, Turkey. The protocol was approved by the Ethics Committee of Ataturk University Medical Faculty Ethical Committee (registration number: 13, date: 26.12.2013) and 70 patients over the age of 60 years with ASA (the classification of the American Society of Anaesthesiologists) III or IV physical status who underwent elective hip replacement surgery between January 1 and May 30, 2014 were included. Before participating in the study, the mental function and confusion states of patients were evaluated using Mini-Mental Status Examination (MMSE) [11]. Patients with cognitive deficit (MMSE lower than 5), under the age of 60 and contraindications to CSA or PCSNB such as coagulation disorder and infection at the puncture site were excluded from the study. Written informed consent was obtained from all participating patients. Patients were informed about the Visual analogue scales (VAS) before surgery. The age, weight and height of the patients, ASA physical status, preoperative electrocardiogram findings, mean arterial blood pressure (MBAP), heart rate (HR) and oxygen saturation values and the presence of additional disease such as hypertension (systolic/diastolic tension >160/95 mmHg) and coronary artery disease were recorded. Thromboprophylaxi was provided using a low dose (40 mg) of low molecular weight heparin (Clexane®, Aventis Intercontinental, France) 12 hours prior to surgery for all patients. Before transfer to the operating room, patients were assigned either to the CSA group (n =35) or to the PCSNB group (n =35) using a computer generated random number by an anesthesiologist responsible for patient allocation. Ringer’s lactate solution was given intravenously at 1 to 2 mL/kg/hour via 18-gauge cannula in a forearm peripheral vein and standard monitoring included invasive arterial pressure, electrocardiography and pulse oximetry was established in the operating room. All patients in both groups were pre-medicated with intravenous (IV) midazolam (1 mg) before the procedure of anaesthesia. All anaesthesia procedures were performed by two experienced anaesthetists.

In the PCSNB group, psoas compartment block was performed using modified Winnie technique [12]. After lateral decubitus position with the side to be operated uppermost and with the hip and knees flexed was provided for each patient, the skin over the lumbar paravertebral region was prepared by coating with sterile drapes. The 7 MHz ultrasound probe (Esaote, Firenze, Italy) was placed to the area (approximately 3–4 cm lateral and parallel to the lumbar spine). After the L_3_ spinous process was identified, the transverse process of L_3_ was located moving the probe horizontally. Then the local anaesthetic was infiltrated into the skin and an insulated stimulation needle (Stimuplex®––A Needle, 150 mm/20 G, Braun Medical, Melsungen, Germany) connected to a nerve stimulator (Stimuplex®, HNS 11, Braun Medical, Melsungen, Germany) was introduced along the long axis of the ultrasound probe. A current strength for nerve stimulator was 0.5 to 0.8 mA at 1 Hz. The ultrasound guidance needle was slowly advanced under to the posterior part of the psoas muscle and the lumbar plexus was confirmed when ipsilateral quadriceps muscle contraction was observed. Following negative aspiration, the 30 mL of 0.25% bupivacaine with 1:200.000 epinephrine (5 μgr/mL) was injected into the psoas compartment. The spread of local anaesthetic in the psoas compartment was demonstrated by ultrasound.

After psoas compartment block was completed, the sciatic nerve block was applied according to the method described by Karmakar et al. [13]. The sciatic nerve was localised within the sub-gluteal space (the area between the hyper-echoic perimysium of the gluteus maximus and the quadratus femoris muscles). Then a needle with the same characteristics connected to a nerve stimulator delivering a current of 0.5 to 0.8 mA at a frequency of 1 Hz was inserted in the long axis of the ultrasound probe and advanced slowly towards the sciatic nerve. After foot plantar flexion indicating sciatic nerve stimulation was observed, 20 mL of the same anaesthetic solution was administered to the sciatic nerve following negative aspiration. The patient was then turned to the supine position and iliac crest block, with 5 mL of the same local anaesthetic solution was performed [14].

In the CSA group, CSA was performed in the L_3_-L_4_ interspaced with the patient in lateral decubitus position with the side to be operated uppermost after cleaning and draping. The epidural space was identified with a Crawford needle and a 22-G (Spinocath®, B. Braun, Melsungen, Germany) catheter with a 27-G Quincke spinal needle was advanced through the epidural space until cerebrospinal fluid was observed in the catheter. Then, the spinal catheter was advanced 2–4 cm into the intrathecal space and fixed using sterile tape. After the cerebrospinal fluid was aspirated, 2.5 mg isobaric 0.5% bupivacaine was injected manually while the patient was in a supine position.

In both groups, the sensory block level was tested using pinprick tests and the motor block level was evaluated with the Modified Bromage scale (scale 0 = full flexion of foot, knee and hip, i.e. no motor block; scale 1 = full flexion of foot and knee, unable to hip flexion; scale 2 = full flexion of foot, unable to knee and hip flexion; scale 3 = total motor block; unable to foot, knee, and hip flexion) three times with an interval of 5 minutes. Sensory and motor block tests were performed bilaterally to evaluate possible epidural spread of the local anaesthetic. When sensory block (a loss of pin prick sensation) was not reached to the level of T_12_ within 10 minutes in the CSA group, additional 2.5 mg of isobaric bupivacaine 0.5% was administered through the catheter at 5-min intervals by limiting the total dose of 15 mg until a T_12_ level of the sensory block was achieved. When the satisfactory block level was provided, surgery was initiated in both groups. General anaesthesia protocol was administered for patients with three unsuccessful attempts to reach to spinal space in CSA group and for block procedure in PCSNB group. Also, if adequate surgical anaesthesia was not achieved after 30 minutes on patients of both groups, techniques were considered as failure and general anaesthesia protocol was administered for these patients. Continuing infusion of propofol at the speed of 10–50 μg/kg/min was planned for each patient with discomfort during operation. Patients required propofol infusion exceeding 50 μg/kg/min were considered to be unsuccessful blockade. Oxygen was delivered with a face mask and lactated Ringer’s solution (5 mL/kg/h) was administered to all patients during surgery. Colloid solutions and paced red cells when necessary (haematocrit level <30%) were used to treat perioperative blood loss. Patients’ MABP, HR and oxygen saturation values were recorded at the beginning of anaesthesia procedure and surgery, every 5 minutes during surgery and at 1 hour after surgery by an observer who was blinded to study groups. Ephedrine (IV, 10–15 mg) was administered in the case of hypotension (a 30% decrease in systolic blood pressure compared with preoperative values) and atropine (IV, 0.5 mg) was applied when bradycardia (the heart rate <45 beats/minute) was observed. Socio-demographic characteristics (age, body mass index, co-morbidities), the application time of anaesthetic technique (the time between the onset and end of anaesthesia procedure), duration of the block procedure (the time from the start of the anaesthetic procedure to the development of full motor block), duration of surgery (the time from the start of the surgical incision to the completion of surgery), highest sensory block level, the amount of intraoperative blood loss (weighing the sponges used during surgery plus the amount of blood in the suction bottle), anaesthetic complications and the number of patients required analgesics and sedatives during the block and surgical procedure were recorded. All surgical procedures were performed by the same three surgeons using the same surgical technique.

After surgery, patients with intensive care requirements were transferred to the intensive care unit (ICU) and patients with stable clinical status were transferred to the orthopaedic ward. An anaesthesiologist blinded to group allocation visited the patients and postoperative side effects such as nausea, vomiting and bradycardia were recorded. Also, postoperative pain was evaluated at rest using a 10-cm VAS (0 cm = no pain; 10 cm = worst pain possible) and pain scores were recorded at 30 min and 1^st^, 2^nd^, 4^th^, 6^th^, 12^th^ and 24^th^ hours post-operatively.

In the CSA group, morphine of 200 μg was administered through the subarachnoid catheter at the end of surgery for postoperative analgesia; the spinal catheter was removed two hours after completion of surgery. The presence of PDPH (increased pain intensity upon standing up from a supine position) was questioned in patients of the CSA group postoperatively. In the PCSNB group, morphine (0.1 mg/kg, subcutaneously) was administered to the patients at the end of surgery to provide postoperative analgesia. In the case of VAS >3 in both groups, rescue analgesia was provided with IV tramadol 50 mg. The reversal time of the motor block and the number of patients required rescue analgesic in groups at first 24 hours postoperatively were recorded. On the postoperative first day, the MMSE was completed to detect the presence of postoperative confusion and the results were compared with the preoperative values.

With hemodynamic parameters as the primary outcome measurement, power calculation analysis revealed 31 patients in each study group to be necessary to find a difference of 20% in comparison with baseline MABP values with a power of 80%, α of 0.05 and β of 0.20 [15]. Data was analysed using SPSS software 12.0 (SPSS Inc., Chicago, IL, USA) and calculated as mean ± standard deviation, P <0.05 was considered significant. The Kolmogorov-Smirnov test was used to assess the normal distribution of data. If data were not normally distributed, comparisons were determined using Mann–Whitney U-test. Comparison of variables at different times between groups such as motor block were conducted using repeated measures two-way ANOVA test and Fisher’s exact test was used to compare the percentage values.

## Results

**Figure 1:**
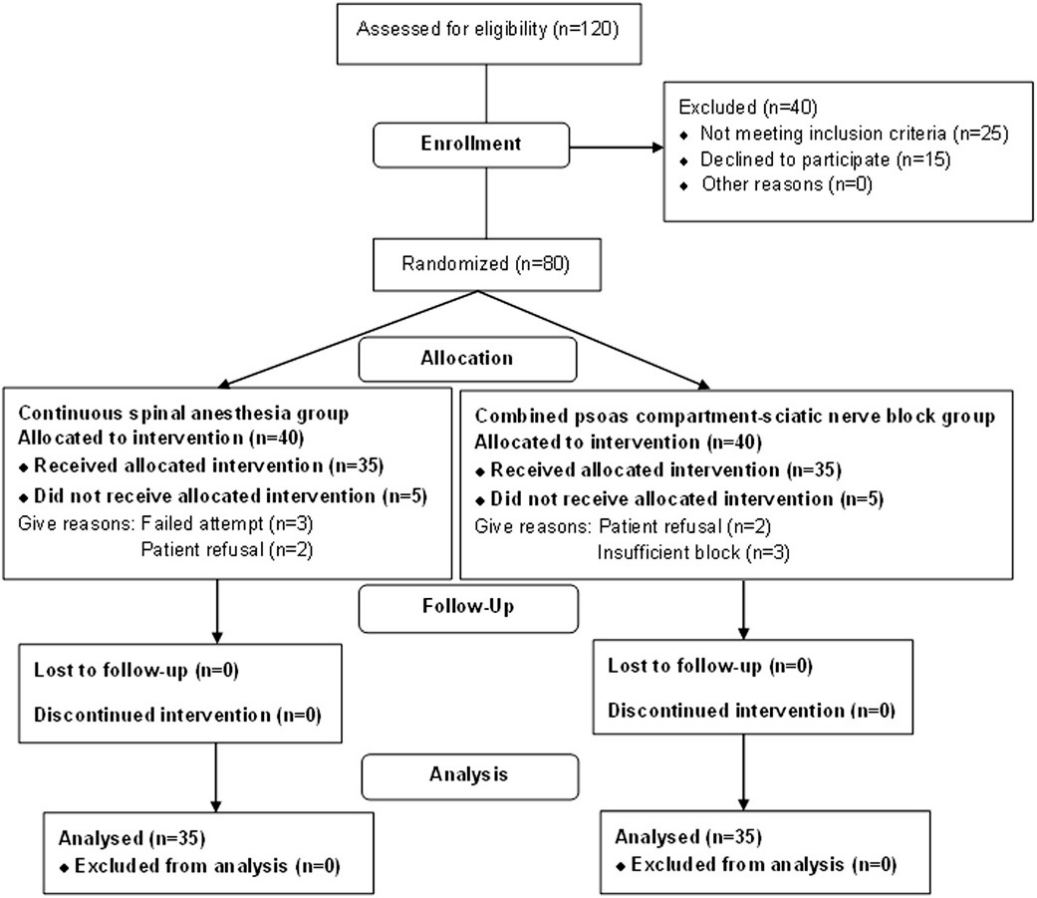
**CONSORT flow diagram.** The course of patients through this study was shown.

Eligible patients for this study were analysed for the primary outcomes and are shown in the CONSORT flow diagram (Figure 1) [16]. Eighty patients were randomly divided into two groups of 40 each. Three patients in both groups required general anaesthesia due to failed or insufficient block, and these patients were excluded from the study.

Clinical characteristics of the patients in both groups were comparable (Table 1). Comorbiditiy was evaluated using the Charlson comorbidity index (CCI) [17] and individual comorbidities were presented in Table 1. Duration of the block procedure (minutes) was significantly shorter in the CSA group (18.51 ± 1.82) than in the PCSNB group (35.54 ± 5.51) (P <0.001). The median level of sensory block was T_9_ in the CSA group and it was L_2_ (range: L_1_-S_2_) in the PCSNB group (Table 2). No patient had fentanyl propofol and midazolam requirements during surgery in the CSA group. In CSA group, total cumulative dose of isobaric bupivacaine per patient (mean ± SD) was 7.01 ± 1.89 mg. All patients in PCSNB group needed continuing infusion of propofol at the speed of 10–50 μg/kg/min during operation. Total intraoperative blood loss (mL) was found to be lower in the CSA group compared with the PCSNB group (283.14 ± 68.66 and 329.57 ± 53.66) (P =0.02) (Table 2). The number of patients requiring rescue analgesic postoperatively was significantly higher in the PCSNB group compared to the CSA group (P =0.0001) (Table 2). There are no difference between preoperative and postoperative MMSE scores (mean ± SD) in both groups (18.85 ± 4.00 ∞18.37 ± 4.37, for CSA group; 19.28 ± 3.80 ∞ 18.57 ± 4.08, for PCSNB group).

**Table 1:** **Clinical characteristics of the patients in both groups**

	CSA group (n = 35)	PCSNB group (n = 35)
Age (years)	73.34 ± 6.31	72.94 ± 7.55
Weight (kg)	69.69 ± 14.01	74.4 ± 15.78
Height (cm)	164.80 ± 7.21	165.77 ± 5.91
Female/Male	17/18	14/21
ASA III/IV	26/9	27/8
LV ejection fraction (%)	40.71 ± 4.71	40.28 ± 4.68
Charlson comorbidity index	4.54 ± 0.85	4.37 ± 1.00
Additional diseases n (%)		
Hypertension	20 (57.1)	22 (62.8)
Diabetes Mellitus	10 (28.5)	12 (34.2)
Coronary heart disease	9 (25.7)	11 (31.4)
Chronic renal failure	2 (5.7)	3 (8.5)
Chronic obstructive pulmonary disease	4 (11.4)	3 (8.5)
Cerebrovascular disease	2 (5.7)	1 (2.8)

Results expressed as mean ± SD or n (%).

**Table 2:** **Anaesthetic characteristics in groups**

	CSA group (n = 35)	PCSNB group (n = 35)	P value
The application time of the anaesthetic technique (minutes, mean ± SD)	9.37 ± 1.72	13.91 ± 3.91	0.001
Duration of the block procedure (minutes, mean ± SD)	18.51 ± 1.82	35.54 ± 5.51	0.001
Duration of surgery (minutes, mean ± SD )	101.37 ± 25.10	103.60 ± 17.48	>0.05
Intraoperative total blood loss (ml, mean ± SD )	283.14 ± 68.66	329.57 ± 53.66	0.02
Maximum sensory level [median (min-max)]	T_9_ (T_6_-T_11_)	L_2_ (L_1_-S_2_)	
The number of patients requiring ephedrine	13	4	0.012
The number of patients requiring rescue analgesics in the first 24 hours postoperatively	11	30	0.0001
Total bupivacaine consumption (mg)	8.50 ± 1.24		

Results expressed as mean ± SD or n.

**Figure 2:**
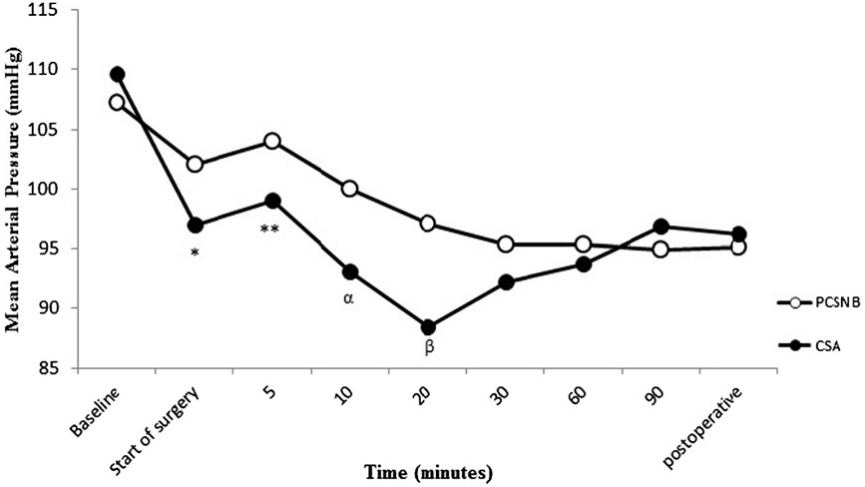
**Mean arterial blood pressure values of patients in groups.** Baseline: Before anaesthesia procedure. Postoperative: One hour after surgery. *P =0.038, **P =0.029, ^α^P =0.012, ^β^P =0.009; compared with PCSNB group. Patients in both groups had significantly lower MABP values during surgery compared with preoperative values (P <0.001).

**Figure 3:**
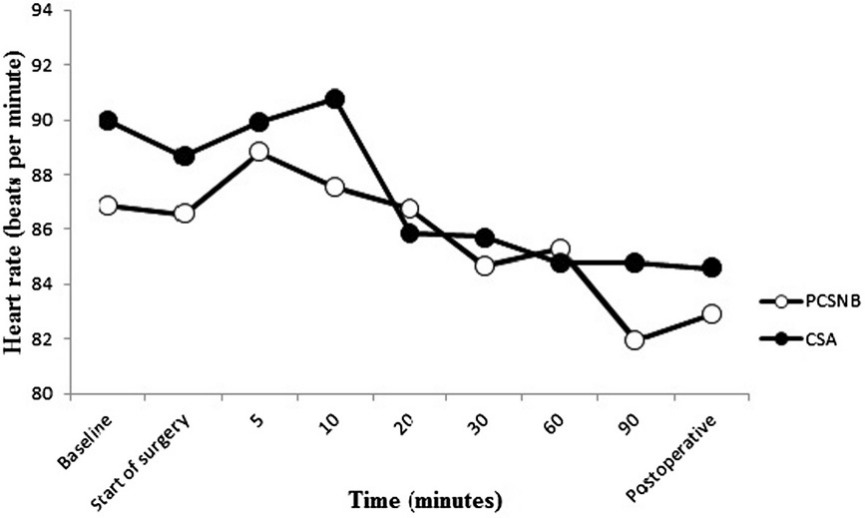
**Heart rate values of patients in groups.** Patients in both groups had similar heart rate values at the beginning of surgery, any time points during surgery and postoperatively (P >0.05).

**Figure 4:**
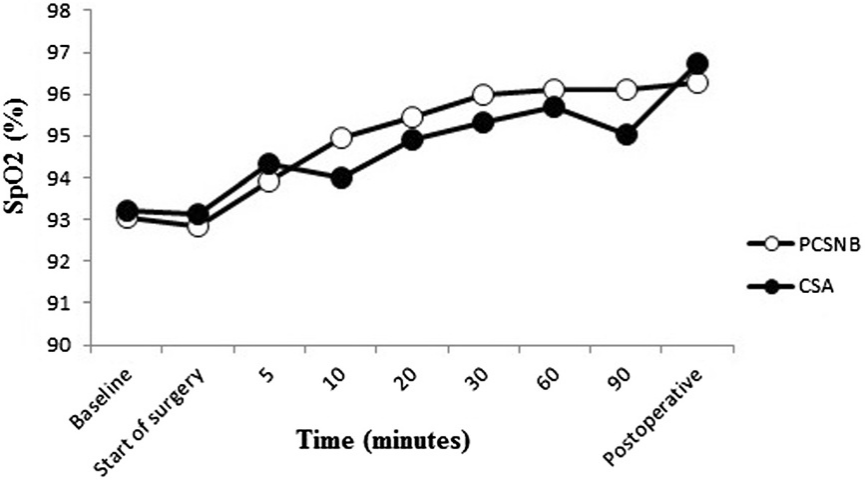
**Peripheral oxygen saturation values of patients in groups.** There were no statistically significant differences between groups in terms of peripheral SpO2 (P >0.05).

The PCSNB group had significantly higher MABP values at the beginning of surgery and at 5^th^, 10^th^ and 20^th^ minutes of surgery compared to the CSA group (P =0.038, P =0.029, P =0.012, P =0.009 respectively). Patients in both groups had significantly lower MABP values during surgery compared with preoperative values (P <0.001) (Figure 2). Arterial hypotension required ephedrine treatment was observed in 13 patients in the CSA group and four patients in the PCSNB group (P =0.012). No patient had PDPH in the CSA group until discharged. In the post-operative period, no patients in both groups had cardiovascular complications. There were no significant differences between groups in terms of HR and peripheral oxygen saturation (SpO2) values during surgery (P >0.05) (Figures 3 and 4). Patients in both groups had similar HR values during surgery compared with preoperative values (p > 0.05).

## Discussion

In this study, we first compared the hemodynamic effects of PCSNB with CSA in elderly high-risk patients undergoing hip replacement surgery. All patients studied had low ejection fraction and CCI scores above 4. Both techniques produced satisfactory quality of anaesthesia in elderly high-risk patients with fewer hemodynamic changes in PCSNB cases compared with CSA cases.

Regional anaesthesia techniques are usually preferred in elderly patients due to the some advantages such as the maintenance of cardiovascular stability and early postoperative mobilization [3]. It is shown that CSA provides less nausea and vomiting, better postoperative analgesia and better hemodynamic stability during anaesthesia induction than single-shot spinal anaesthesia combined with morphine patient-controlled analgesia after total hip replacement surgery [18]. This technique also provides extended analgesia allowing the administration of repeated local anaesthetics according to the patient’s needs during surgery and ensures cardiovascular stability [7]. Conversely, PCSNB provides minimal hemodynamic effects without causing a reduction in the regional blood flow of the extremity [8].

The use of large needles and catheters for CSA was found to be associated with PDPH [6]. So, microcatheters have been developed and lower incidence of PDPH was reported following the use of microcatheters for CSA [7]. On the other hand, the presence of case reports of cauda equina syndrome led to the restriction of the use of spinal micro-catheters in the United States and Australia [19, 20]. But, studies showed that the development of this syndrome is associated with neurotoxic effects and poor distribution of local anaesthetics rather than micro-catheters [21, 22]. So, micro-catheter for CSA is widely used in Europe for lower limb surgery. In this study, a catheter over needle was used and PDPH or cauda equina syndrome was not observed in any patient. Similar to our results, Kılınc et al. [23] reported no PDPH or cauda equina syndrome in elderly patients undergoing hip surgeries using the anaesthesia technique of CSA via an over-the-needle catheter.

In this study, the application time of the anaesthesia technique was longer in the PCSNB group than those of the CSA group. Our findings were consistent with the findings of Adalı et al. [24]. We reported that PCSNB is more suitable than CSA with regard to hemodynamic changes for hip replacement surgery in elderly high-risk patients (11% of the patients had a MAP decrease over 30% in the PCSNB group and 37% in the CSA group). This high frequency of blood pressure changes were probably related to existing additional co-morbidities in our patients. Also, the median level of sensory block was T_9_ in the CSA group and it was L_2_ in the PCSNB group. This high level of sensory block may be reason for hypotension and more ephedrine requirement in CSA group. Unlike our results, Casati et al. [25] reported similar hemodynamic side effects between spinal anaesthesia and combined sciatic-femoral nerve block techniques in outpatients receiving knee arthroscopy. Also, Adalı et al. [24] found similar hemodynamic changes between spinal anaesthesia and combined sciatic/lumbar plexus nerve block techniques in patients undergoing lower extremity orthopaedic surgery. The reason for these differences may be due to the differences in the clinical characteristics of the patients selected for this study. While this study was performed among elderly patients with ASA III or IV, the above studies [24, 25] were performed among patients with ASA I or II. Conversely, de Leeuw et al. [26] reported a significant increase in the heart rate during and after the PCSNB nerve block procedure compared with baseline values in patients undergoing total hip arthroplasty revision surgery. Also MAP values showed a significant increase during block procedure and a significant decrease during the post-block period in their study. In this study, patients in both groups had similar heart rate and MAP values during surgery compared with baseline values. However, MAP values at the 5^th^, 10^th^ and 20^th^ minutes of surgery were higher in the PCSNB group compared to the CSA group. Also, the number of patients requiring ephedrine administration was higher in the CSA group compared with the PCSNB group.

Life threatening complications such as systemic toxicity, retroperitoneal hematoma and renal puncture may occur following a PCSNB procedure. We excluded patients with coagulation disorders from the study and the PCSNB block procedure was performed using a nerve stimulation technique and under ultrasound guidance by observing the extent of slowly administered local anaesthetic agent. For these reasons, no major complications due to the PCSNB block procedure were observed in any of our patients intra-postoperatively. Indeed, the use of anticoagulant or anti-platelet drugs [27] and the injection of local anaesthetic with high injection pressure [28] were found to be associated with an increase in undesirable complications during nerve block procedure. Also, the application of the blocks under ultrasound guidance reduced the incidence of complications in the post-block period [29, 30]. We did not observe any serious complication of anaesthesia in the CSA group similar to the literature [7, 31].

After hip replacement surgery, there is an increased risk of deep venous thrombosis and pulmonary embolism due to endothelial wall damage with surgical instruments, postoperative immobilization and increased coagulation [32]. It was reported that the prevalence of deep venous thrombosis is over 50% following total knee or hip replacement surgery in cases not receiving thromboprophylaxis [33]. All patients in this study received thromboprophylaxis with low molecular weight heparin and we did not observe any deep venous thrombosis or pulmonary embolism clinically in cases during or after surgery.

Perioperative blood loss was lower in the CSA group than those of the PCSNB group in this study. Stevens et al. [34] reported that posterior lumbar plexus block provides a reduction operative and postoperative (48 hours) blood loss attenuating sympathetic tone in medium and small vessels around the hip joint in patients undergoing total hip arthroplasty. On the other hand, CSA leads to a decrease in total peripheral resistance and arterial blood pressure as a result of sympathetic blockade. Blood in the operative field spreads to the other tissues due to the fall in arterial pressure, so causes the intraoperative blood loss reduction in the CSA technique [35].

In this study, PCSNB was successful in all patients, but surgical anaesthesia was not achieved in 7.8% of the cases. However in group CSA, catheter insertion was unsuccessful in 3 patients (7.8%), so surgery was completed under general anaesthesia in three patients in both groups. Thus, both CSA and PCSNB techniques provided adequate anaesthesia in the majority of patients (92.1% for PCSNB, 100% for CSA) in our study. Similar to our results, de Visme et al. [8] reported that plain bupivacaine spinal anaesthesia and combined lumbar/sacral plexus block provides adequate anaesthesia for repair of hip fracture in patients over 85 years of age. Adalı et al. [24] also showed that both spinal anaesthesia and combined sciatic/lumbar plexus nerve block are effective in lower extremity orthopaedic surgeries.

Based on our clinical experiences in elderly patients with comorbidities, we used a starting low dose of isobaric bupivacaine and we administered it gradually to achieve better hemodynamic stability in patients of CSA group. The success rate of CSA providing surgical anaesthesia was achieved in 100% of patients in our study. Indeed, Paqueron et al. [36] studied the characterization of onset and duration of peripheral nerve block in a population of elderly patients and they showed a positive relationship between age and duration of complete sensory and motor blockade. Also, Benzon et al. [37] reported that smaller doses of local anaesthetic are required clinically for anaesthesia in older age groups. On the other hand, Kroin et al. [38] reported longer duration of sciatic nerve block with local anaesthetics in diabetic rats compared with non-diabetic rats. Our study populations had multiple co-morbidities and 31% of our patients were diabetic. Eventually, our high success rate of CSA with low dose local anaesthetic may be explained with the presence of existing co-morbidities and advanced age in our study populations.

This study was performed in a relatively small population of patients. This was a limitation for this study.

## Conclusions

This is the first study in the literature comparing the CSA technique with the PCSNB technique with regards to hemodynamic effects in elderly high-risk patients. CSA and PCSNB produce satisfactory quality of anaesthesia in elderly high-risk patients with fewer hemodynamic changes in PCSNB cases compared with CSA cases. However, there is a significantly longer application time of anaesthetic technique and duration of the block procedure in the PCSNB group. Further studies consisting of a greater number of patients are required to evaluate the effects of PCSNB and CSA on hemodynamic and anaesthetic parameters in elderly high-risk patients.

## Abbreviations

PCSNB: Combined psoas compartment-sciatic nerve block
CSA: Continuous spinal anaesthesia
PDPH: Post-dural puncture headache
MAP: Mean arterial blood; pressure
HR: Heart rate
ASA: American Society of Anaesthesiologists
MMSE: Mini-Mental Status Examination.
